# Incidence and outcomes of acute kidney injury following orthotopic lung transplant: a population-based cohort study

**DOI:** 10.1186/cc13577

**Published:** 2014-03-17

**Authors:** P Fidalgo, M Ahmed, SR Meyer, D Lien, J Weinkauf, FS Cardoso, K Jackson, SM Bagshaw

**Affiliations:** 1 University of Alberta, Edmonton, Canada; 2 Alberta Health Services, Edmonton, Canada

## Introduction

Acute kidney injury (AKI) is a serious complication following lung transplantation (LTx) [1-3]. We aimed to describe the incidence and outcomes associated with AKI following LTx.

## Methods

A retrospective population-based cohort study of all adult recipients of LTx at the University of Alberta between 1990 and 2011 was performed. The primary outcome was AKI, defined and classified according to the Kidney Disease: Improving Global Outcomes (KDIGO) criteria, in the first seven postoperative days. Secondary outcomes included risk factors, utilization of renal replacement therapy (RRT), occurrence of postoperative complications, mortality and kidney recovery.

## Results

Of 445 LTx recipients included, AKI occurred in 306 (68.8%), with severity classified as stage I in 38.9% (*n *= 173), stage II in 17.5% (*n *= 78) and stage Ill in 12.4% (*n *= 55). RRT was received by 36 (8.1%). Independent risk factors associated with AKI included longer duration of cardiopulmonary bypass (per minute, odds ratio (OR) 1.003; 95% Cl, 1.001 to 1.006; *P *= 0.02), and mechanical ventilation (per hour (log-transformed), OR 5.30; 95% CI, 3.04 to 9.24; *P *< 0.001), and use of cyclosporine (OR 2.03; 95% CI, 1.13 to 3.64; *P *= 0.02). In-hospital and 1-year mortality were significantly higher in those with AKI compared with no AKI (7.2% vs. 0%, adjusted *P *= 0.001; 14.4% vs. 5.0%, adjusted *P *= 0.02. respectively). At 3 months, those with AKI had greater sustained loss of kidney function compared with no AKI (estimated glomerular filtration rate (mean (SD)) 68.9 (25.7) vs. 75.3 (22.1) ml/minute/1.73 m^2^, *P *= 0.01).

## Conclusion

By the KDIGO definition, AKI occurred in two-thirds of patients following LTx. AKI portended greater risk of death and loss of kidney function.
